# Low-Pressure Microwave Plasma Reduction of Iron and Copper Salt Compounds at Low Temperatures for Oxidation State Alteration and Functional Applications

**DOI:** 10.3390/ma16083221

**Published:** 2023-04-19

**Authors:** Mirco Weber, Anna Scheglov, Wiebke Dörries, Johann Benedikt Meyer, Wolfgang Viöl

**Affiliations:** 1 Faculty of Engineering and Health, HAWK University of Applied Sciences and Arts, Von-Ossietzky-Straße 98/100, 37085 Göttingen, Germany; mirco.weber@hawk.de (M.W.); anna.scheglov1@hawk.de (A.S.); wiebke.doerries@hawk.de (W.D.); 2 Institute of Inorganic Chemistry, Georg August University of Göttingen, Tammannstraße 4, 37077 Göttingen, Germany; j.meyer01@stud.uni-goettingen.de; 3 Application Center for Plasma and Photonics, Fraunhofer Institute for Surface Engineering and Thin Films, Von-Ossietzky-Straße 100, 37085 Göttingen, Germany

**Keywords:** microwave plasma, low-pressure plasma reduction inclusion, metal salt films, reduction strategy

## Abstract

The influence of plasma-reduction treatment on iron and copper compounds at different oxidation states was investigated in this study. For this purpose, reduction experiments were carried out with artificially generated patina on metal sheets and with metal salt crystals of iron(II) sulfate (FeSO_4_), iron(III) chloride (FeCl_3_), and copper(II) chloride (CuCl_2_), as well as with the metal salt thin films of these compounds. All the experiments were carried out under cold low-pressure microwave plasma conditions; the main focus was on plasma reduction at a low pressure in order to evaluate an implementable process in a parylene-coating device. Usually, plasma is used within the parylene-coating process as a supporting tool for adhesion improvement and micro-cleaning efforts. This article offers another useful application for implementing plasma treatment as a reactive medium in order to apply different functionalities by an alteration in the oxidation state. The effect of microwave plasmas on metal surfaces and metal composite materials has been widely studied. In contrast, this work deals with metal salt surfaces generated from a solution and the influence of microwave plasma on metal chlorides and sulfates. While the plasma reduction of metal compounds commonly succeeds with hydrogen-containing plasmas at high temperatures, this study shows a new reduction process that reduces iron salts at temperatures between 30 and 50 °C. A novelty of this study is the alteration in the redox state of the base and noble metal materials within a parylene-coating device with the help of an implemented microwave generator. Another novelty of this study is treating metal salt thin layers for reduction purposes in order to provide the opportunity to include subsequent coating experiments to create parylene metal multilayers. Another new aspect of this study is the adapted reduction process of thin metal salt layers consisting of either noble or base metals, with an air plasma pre-treatment prior to the hydrogen-containing plasma-reduction procedure.

## 1. Introduction

Metals and metal compounds play an important role in both nature and technology due to their versatile properties, their different oxidation states, and the various types in which they occur. Iron, for example, fulfils its enzymatic role as Fe^2+^ in a heme structure in catalases and peroxidases, in which it varies its oxidation state in the course of enzyme reactions [1]. These variations cause different functionalities and reactivities. Therefore, the control of the oxidation state is of great importance within functional materials and coatings. However, sometimes the required oxidation state is unstable or metastable under atmospheric conditions. For example, the Fe^3+^ state is mostly preferred to the Fe^2+^ state under oxygen-containing atmospheres because Fe^3+^ has a 3d^5^ configuration. This means that the five 3d orbitals are half occupied, which provides the ions with a stability advantage over the 3d^6^ occupation of Fe^2+^. The latter has a 3d^6^ configuration that corresponds to neither full occupation nor half occupation and, thus, to a stable electron configuration. Copper also has several prominent oxidation states, including Cu^+^ and Cu^2+^. Of these states, Cu^+^ has the most stable orbital configuration with 3d^10^, as the 3d orbitals are fully occupied, while Cu^2+^ is only 3d^9^-configured. However, Cu^2+^ compounds are generally more abundant since the enthalpy of the hydration of 2121.29 kJ/mol (21.99 eV/atom) is much larger than that of Cu^+^, which is 581.58 kJ/mol (6.03 eV/atom). This greater abundance contributes more energy than that of orbital stabilization⁠ [2].

Since the most prominent oxidation states of iron and copper are directly adjacent to each other with Fe^2+^/Fe^3+^ and Cu^+^/Cu^2+^, they are ideal candidates for the exploration of a stepwise or complete reduction in low-pressure plasma. Only a single electron per ion needs to be taken up for a staggered change in the oxidation state. This greatly facilitates the transition from stage to stage compared to the stage change required for the expenditure of several electrons per process.

Metallic interlayers in thin films are often used for electrical signal transmission by barrier layers [3,4]. Metals in these low-layer thicknesses are difficult to process, so they are usually deposited via sputter processes.

Many metal deposits from sputter electrodes—especially those with low substrate quantities—are not optimally deposited for the layer system by such sputter processes; instead, they are separated as residues within the sputter system. Furthermore, homogeneous sputter deposition is preferably carried out under a low pressure, which causes the process control to be time consuming with higher costs, making the entire coating process more complex. Additional methods, such as e-beam or ion-beam deposition, also require high amounts of energy and increased costs.

Since electronic components and medical implants consist of a material composite with parylene layers and metal thin films [5,6,7,8,9,10,11], the effect of a microwave plasma on different metal salts based on copper and iron within a parylene-coating system is explored in this article. The application of transition metals in one of their oxidized forms as thin films can also be of benefit, including the creation of specific electrical properties in a material composite [12]. Plasma is a useful tool for creating metal compounds of a small size. For example, Myochi et al. reported a TaC nanoparticle synthesis that was achieved by an arc plasma of tantalum and methane [13]. Co_3_O_4_ nanoparticles could also be synthesized by using microplasmas from a cobalt foil as an anode material [14]. In general, the plasma-assisted synthesis of metallic nanoparticles plays a major role, as both the reaction conditions and the reaction time can be gentler than they are in conventional wet-chemical synthesis methods [15].

Plasma can also be used for direct synthesis [16,17,18,19,20,21]. For this reason, this study deals with the plasma-assisted modification of the oxidation state of thin metal salt layers, which can be applied from a solution in a relatively straightforward manner.

Various process gases can be used to oxidize metallic surfaces by increasing the oxidation number, as Shin et al. [22] demonstrated with their modification of aluminum surfaces. In their study, microwave plasma jets with Ar, Ar/O_2_, and Ar/H_2_, with the proportion of oxygen and hydrogen of 1.5%, were used as the process gas. The contact angle measurements showed that plasma with Ar/H_2_ as the process gas mixture caused the strongest changes in the metal surfaces. In another study, Sabat et al. [23] presented the following reaction mechanism for plasma reduction via hydrogen plasma:

MOx + xH_2(g)_ → M + xH_2_O_(g)_ (ΔG_1_ > 0)

Hydrogen Plasma: H_2(g)_ → 2H/2H^+^/2H^2+^/2H^3+^/H_2_* (ΔG_2_ >> 0)

MOx + xHydrogen Plasma → M + xH_2_O(g) (ΔG_3_ = ΔG_1_ − xΔG_2_ < 0)

It was discovered that reduction with atomic hydrogen was much more effective than it was with molecular hydrogen. For example, the successful reduction of Fe_2_O_3_ with molecular hydrogen took place at a temperature of 310 °C, while it took place at 40 °C when atomic hydrogen was used as the process gas.

In a study on the reduction of chromium and arsenic ions, Chandana et al. [24] presented a possibility of using a cold plasma source to reduce these two substances in aqueous solution through the formation of reactive species, such as H_2_O_2_, OH radicals, hydrated electrons (according to Goodman’s method attached to chlorine radicals to form a chloride anion), and hydrogen radicals. Reductive layer modification was also successfully demonstrated using silver and palladium salt layers.

For this purpose, Crowther et al. [25] generated a hydrogen glow discharge over one silver nitrate and one palladium acetate layer on a glass slide and subsequently demonstrated a successful reduction. Here, the diffusion of atomic hydrogen turned out to be the key element for the reduction’s success. Cui et al. [26] explored a way to generate elemental silver within a polyimide film. To do this, silver nitrate was introduced into the polyimide material and reduced at a low pressure with a glow discharge, creating a polyimide film with silver nanoparticles. A brief overview of the key messages of this introduction is shown in Table 1.

In this article, the reduction possibilities using the plasma of oxidized metallic compounds within a parylene low-pressure coating system are evaluated in order to compare the different possibilities of modifying metal-based layers for parylene–metal multilayer coating projects [27]. The study attempts to adapt the previous procedures of oxidation state alteration via plasma technology to a low-pressure coating device. It is also of great importance to evaluate the plasma influence with respect to its reduction impact on metal salt layer materials, which, to the best of our knowledge, has not yet been the focus of plasma reduction studies. The plasma processes in this publication were performed at low temperatures. This circumstance is a novelty as well because a high process temperature is usually chosen to reduce base metals such as iron.

## 2. Materials and Methods

The low-pressure plasma experiments were carried out using the LAB Coater 300 LV 35 RR (Plasma Parylene Systems GmbH, Rosenheim, Germany). This system consists of a coating unit with an integrated evaporation and pyrolysis unit, as well as a coating chamber equipped with a microwave generator. The latter can ignite low-pressure plasma with pressures of 80 to 90 Pa for ambient air and 170 to 180 Pa for Varigon treatment inside the coating chamber with temperatures between 30 and 50 °C. In addition to the coating unit, the system also has a cold trap with a connected rotary vane vacuum pump to generate the vacuum required for coating and plasma treatment.

Several different process gases were used for the plasma treatments. Ambient air from the laboratory was the oxidative process gas for the oxidation experiments. While Varigon gas (Linde AG, Hannover, Germany), consisting of 95% argon and 5% hydrogen, was the hydrogen-containing gas at a low pressure.

With ambient air as the process gas, the low-pressure plasma treatments were carried out at pressures between 80 and 90 Pa, a microwave frequency of 2.45 GHz, and a microwave power of 350 W. In the Varigon experiments, the plasma treatments were performed at pressures between 170 and 180 Pa, a microwave frequency of 2.45 GHz, and a microwave power of 850 W. The power density was 8.75 W/L for the plasma treatments with ambient air and 21.25 W/L for the experiments with Varigon gas. Using internally installed mass flow controllers, the gas flow rate was set to 300 sccm (standard cubic centimeter per minute) for ambient air and 1000 sccm for Varigon.

The metal sheet strips were pre-cleaned with acetone and isopropanol and then abraded on the surface with an abrasive fleece to remove rolling oil residues and any passivation layers. CuCl_2_ · 2 H_2_O, FeCl_3_ · 6 H_2_O, and FeSO_4_ · 7 H_2_O were purchased from the company Carl Roth GmbH + Co. KG (Karlsruhe, Germany) as salt materials for crystal experiments and metal salt thin-layer synthesis.

Artificially oxidized patina films were produced on the metal sheets with semi-concentrated HNO_3_ for the copper strips and with 25% acetic acid from Carl Roth GmbH + Co KG for the steel strips. For this purpose, a drop of the respective acid was dripped onto the metal sheets. Afterward, they were allowed to react for 18 h, enabling a patina to form and liquid residues to dry, for the most part, on the surface.

X-ray photoelectron spectroscopy (XPS) measurements to investigate the chemical composition of the metallic layers were taken using the PHI 5000 Versaprobe II (Ulvac-phi Inc., Osaka, Japan) with a lanthanum hexaboride filament and aluminum anode. The optical inspection of the layers was performed with a digital microscope (VHX 6000 from Keyence Corp., Neu-Isenburg, Germany).

The XPS samples were prepared with metal salts dissolved In methanol/distilled water (*β* = 12.8 g/L) on round microscope coverslips (Ø = 10 mm). The pH values of these solutions were measured with a Five Easy pH meter (Mettler Toledo, Columbus, OH, USA). The CuCl_2_ solution had a pH value of 1.74, and the two iron salt solutions showed values of 1.20 for FeCl_3_ and 2.01 for FeSO_4_. For XPS measurements, 10 drops of the metal salt solution were applied to the cover glasses and dried under a low pressure at approx. 5 Pa. After the drying time, a uniformly covering metal salt film had formed. Methanol was chosen as the solvent because it has a lower boiling point than water. This has the advantage that the solvent is easier to remove under low-pressure conditions, which facilitates the creation of metal salt thin layers. FeSO_4_ was additionally dissolved in distilled water because of the carbon pollution caused by methanol.

## 3. Results

### 3.1. Initial Oxidation and Reduction Plasma Tests with Metal Strips

First, the effect of the microwave plasma inside the coating device on metals had to be investigated. Since copper and iron are widely used in the electrical industry and for catalysis purposes, these two elements were chosen.

**Figure 1:**
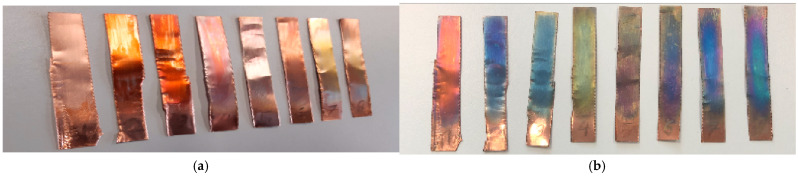
Copper strips directly after oxidative low-pressure plasma treatment (**a**) and after three days (**b**) with pronounced interference effects of the oxidized layers. Picture (**a**) shows the reddish discoloration, which appeared immediately after the ambient air plasma treatment. After one week, different colors appeared, caused by interference effects of copper oxide layers with different thicknesses, as can be seen in photo (**b**).

**Figure 2:**
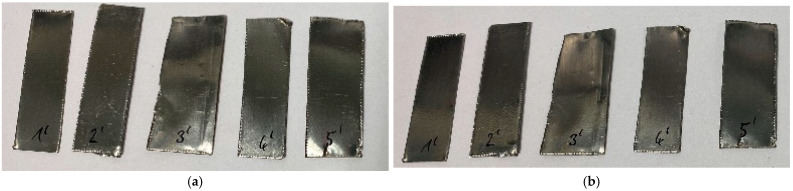
Steel strips before (**a**) and one week after oxidative low-pressure plasma treatment (**b**). The samples on the right-hand picture were treated for 12 (1′), 24 (2′), 36 (3′), 48 (4′), and 60 (5′) min. No plasma effect was visible on the steel strips.

For the experiments, several strips of either copper or steel, each approximately 5 cm to 8 cm long and 2 cm wide, were placed in the coating chamber and treated with air plasma at 120 Pa for 60 min. Immediately after the treatment, only a slight reddish discoloration was visible in the copper strips. After a few days, significant discoloration appeared, probably caused by the interference effects of the thin copper oxide layer (Figure 1). For the second element iron, no changes were visible on the surface, neither immediately following treatment nor one week afterward (Figure 2), suggesting that no significant plasma-induced oxidation had occurred.

**Figure 3:**
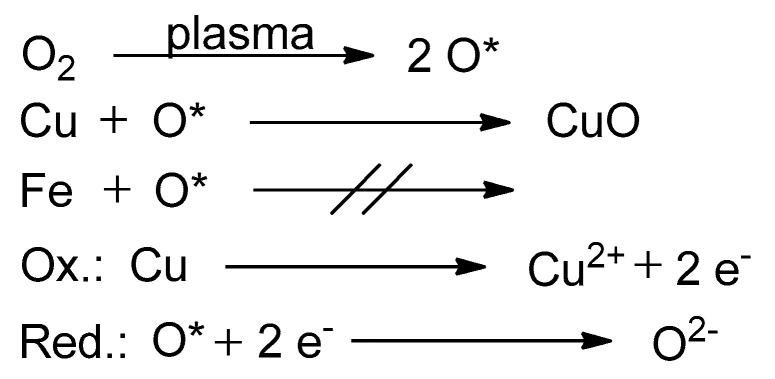
Redox equations of metal oxidation based on the experimental results. The first equation describes a plasma-induced dissociation of molecular oxygen to excited oxygen atoms (O*). The two equations below show the specific oxidation and reduction processes.

A suggested redox equation system can be seen in Figure 3. The plasma affects primarily the process gas, which was in this case ambient air. One essential component for oxidation attempts of this air is oxygen. The effect of plasma is the dissociation of molecular oxygen to its atomic form. Another plasma effect is the excitation of oxygen to higher energetic levels, leading to a more reactive oxygen species. Oxidation success is visible in the case of copper (Figure 1). On the copper strips, an oxidized layer of copper oxide, where copper has the oxidation state of +2, became visible through the interference effects. Copper had therefore lost two electrons, which were taken by oxygen (see Figure 3). As a result, copper was oxidized and oxygen was reduced.

The metal strips were analyzed via XPS after the ambient air plasma treatment on their atomic composition. These results are shown in Table 2.

The ratio between oxygen and metal content decreases after plasma treatment, as can be seen in Table 2. This might be counterintuitive since oxygen-containing plasma should increase this ratio. A possible explanation could be the existence of oil residues from the processing of the sheets, which also contain oxygen. This is indicated by the high carbon amount in the reference samples. Another reason can be found in a specific property of oxygen-containing plasmas, namely that they are known for their etching effects. This could have led to an abrasion of the upper oxygen layer and to the formation of thin metal oxide films, which are responsible for their colorful interference effects, as can be seen in Figure 1b. In conclusion, there are two possible explanations: the first would be plasma cleaning to deplete oil residues on the metal strips and, afterwards, a plasma etching process could have occurred.

**Figure 4:**
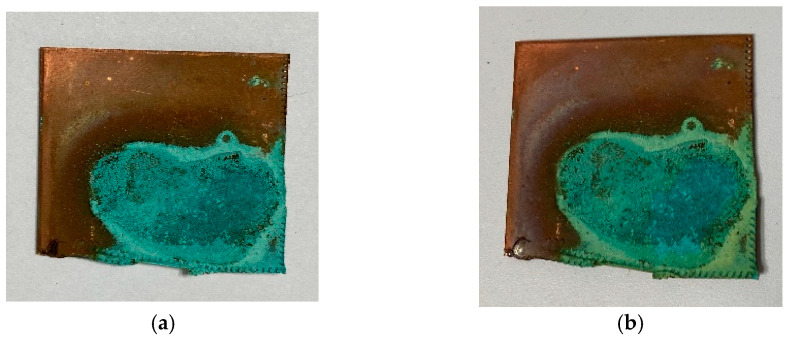
Copper sheets with copper nitrate patina before (**a**) and after reductive low-pressure plasma treatment (**b**). The plasma influence led to a yellowish discoloration of the artificial patina, which picture (**b**) shows.

**Figure 5:**
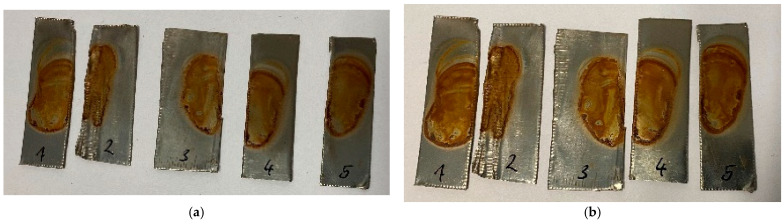
Steel strips after the application of diluted acetic acid (**a**) and directly after reductive low-pressure plasma treatment with Varigon gas for 12 (1), 24 (2), 36 (3), 48 (4), and 60 (5) min (**b**). In comparing these two pictures, no significant change in the artificial patina is visible.

It is also necessary to observe the reduction potential of the low-pressure plasma in order to evaluate the redox impact of this kind of plasma source. To this end, the metal sheet samples were first oxidized on the surface with acid so that an artificial local patina was created (Figure 4 and Figure 5). This patina on the copper sheets consists of copper(II) nitrate, in which the copper exists in the oxidation state of +2. The brown precipitate on the steel sheets is called iron(III) acetate. The oxidized iron has the oxidation number of +3 in this patina. The samples were then treated with microwave-induced low-pressure Varigon plasma for 60 min. As can be seen in Figure 5, no change was observed in the oxidized steel sheet samples after the application of hydrogen-containing low-pressure plasma. After 60 min of plasma exposure, slight changes were detected in the oxidized layer.

**Figure 6:**
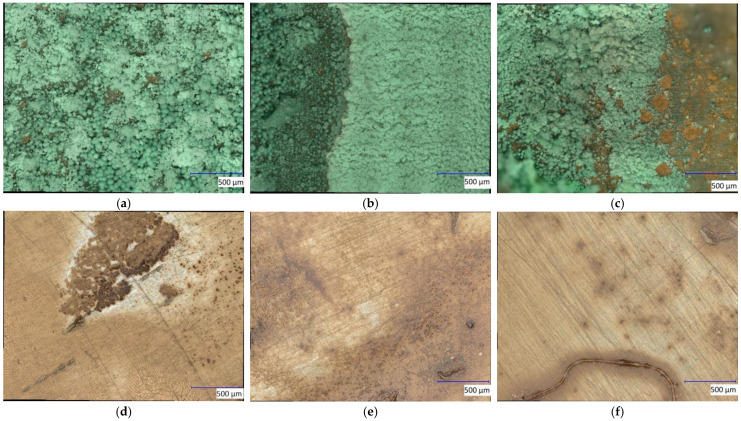
Microscope images of the copper (**a**–**c**) and iron patinas (**d**–**f**) before plasma treatment (**a**,**d**) and after 30 min (**b**,**e**) and 60 min (**c**,**f**) of Varigon plasma at low-pressure. This plasma influence led to a darker discoloration of the copper nitrate patina over the treatment times. The iron acetate patina showed less significant alterations with increasing treatment time. Only a slight grayish color change in the patina can be seen.

It can be concluded from the microscopic observation (Figure 6) that crystalline structures were formed, and that the grey steel background can also be recognized to some extent. However, no significant changes could be detected in the oxidized layer on the steel sheet samples. Thus, even Varigon plasma discharge induced by low-pressure plasma does not seem to enable the sufficient reduction of oxidized iron compounds.

In Figure 4, slight changes could be observed on the copper patina after the treatment period. The thinner parts of the patina turned slightly yellow, resulting in a yellow-green tint in these areas. These color changes suggest that the plasma treatment seems to have an effect on the oxidized copper parts of the patina.

After both 30 min and 60 min of Varigon plasma treatment, dark discolorations were visible on the patina surface. These became even more pronounced with an increasing treatment time, as can be seen in the upper image series of Figure 6 of the copper nitrate patina. These discolorations may be indicative of the formation of copper oxide and copper nitride species. The reason for this transformation could be due to the possible splitting of the nitrate ligands into nitrogen and oxygen under the influence of the plasma, which can contribute to the formation of these species.

To summarize this point, it can already be concluded that the microwave plasma exerts a greater influence on Cu^2+^ ions than on Fe^3+^ ions.

A possible explanation for this would be that in the present iron compounds, Fe^3+^ ions exist in a stable electron configuration. Fe^3+^ is a d^5^ compound, which means that the five d orbitals are each half occupied, which is a criterion for a stable electron configuration. This makes it energetically very costly to change this oxidation state, since with each added electron, the Fe^3+^ ions move further and further away from their energetically most favorable configuration. For the Cu^2+^ compounds, which have a d^9^ configuration, such a stabilized state does not exist since empty, half, or fully occupied orbitals are considered particularly stable. Because of this, it is advantageous to pick up another electron in order to achieve the more stable d^10^ configuration. Therefore, it is easier for a Cu^2+^ compound to be reduced than for a Fe^3+^ compound, since the energetic path to a stable configuration is much shorter [28]. In summary, low-pressure plasma was only able to affect metallic copper surfaces as well as copper nitrate patina, but the latter was effected only slightly.

### 3.2. Reduction of Oxidized Metals in Salt Compounds

Metal salts are suitable for metal thin-film formation because they can be easily applied in a dissolved state. To investigate a better approach to layer application, the focus was placed on various metal salts with characteristic metal oxidation states, such as those that also occur within the patina material on metal strips.

After drying the metal salt solution layer, the oxidation state of the metal compound can be modified by plasma oxidation or reduction, depending on the application goal of the coating material. For this purpose, compounds were chosen that contain a relatively high oxidation state of the elements under investigation. FeSO_4_, FeCl_3_, and CuCl_2_ were selected for this study in this respect. FeSO_4_ also has the advantage that it can be treated both reductively to oxidatively and modify the oxidation state.

**Figure 7:**
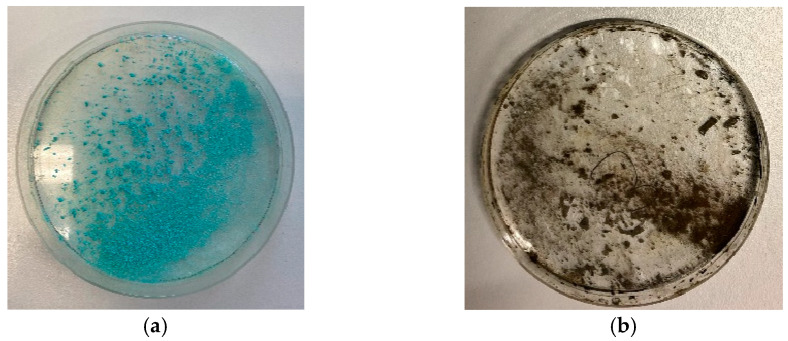
CuCl_2_ · 2 H_2_O crystals before (**a**) and after 30 min reductive Varigon low-pressure treatment (**b**). A dark discoloration as indicator for a strong plasma influence can be seen in picture (**b**).

**Figure 8:**
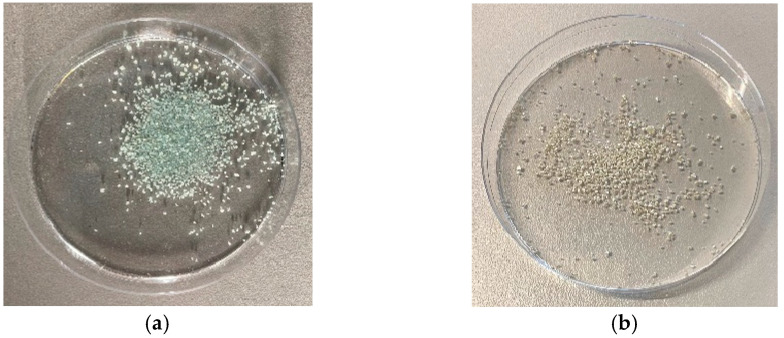
FeSO_4_ · 7 H_2_O crystals before (**a**) and after 30 min reductive Varigon low-pressure treatment (**b**). The plasma-induced color change from a pale turquoise to an almost silver gray can be seen by comparing pictures (**a**,**b**).

**Figure 9:**
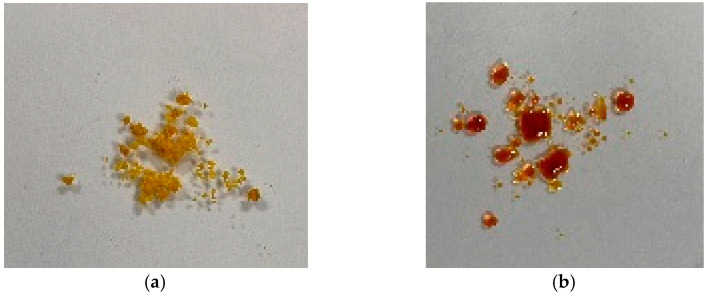
FeCl_3_ · 6 H_2_O crystals before (**a**) and after 30 min reductive Varigon low-pressure treatment (**b**). As can be seen in picture (**b**), the plasma caused a liquefaction of the crystalline starting material.

As can be seen in Figure 7, Figure 8 and Figure 9, the low-pressure Varigon plasma treatment has a recognizable effect on all three salts under investigation. In the case of CuCl_2_, an intensive darkening of the salt to a very dark yellowish-brown, almost appearing black (Figure 7), occurred after 30 min. The plasma treatment of FeSO_4_ did not lead to a darkening of the salt crystals but rather to a significant change in color. After 30 min of plasma treatment, the green crystals had turned a light, almost silvery, metallic gray (Figure 8). No drastic color change occurred with the Varigon treatment of FeCl_3_. Only the yellow-red changed to a more reddish color. However, a deliquescence of the crystals occurred, and a crystalline liquid was formed (Figure 9).

**Figure 10:**
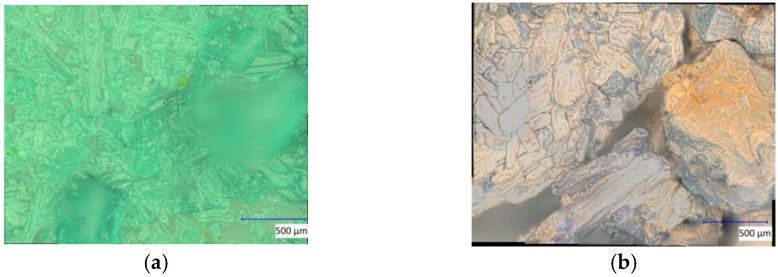
Microscope images of CuCl_2_ crystals before (**a**) and after the one-hour Varigon plasma treatment at a low pressure (**b**). Picture (**b**) shows the plasma-induced color change as an indicator of a surface alteration caused by plasma reduction processes.

**Figure 11:**
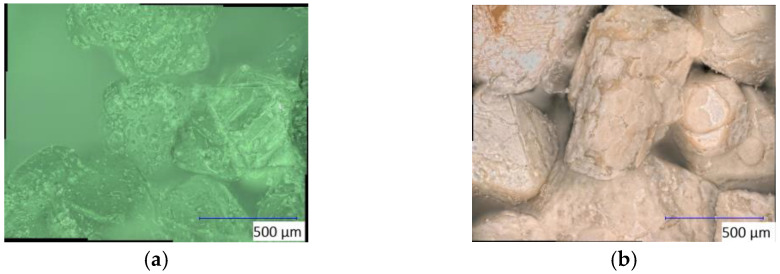
Microscope images of FeSO_4_ crystals before (**a**) and after the one-hour Varigon plasma treatment at low pressure (**b**). The comparison of the two pictures shows a clear discoloration, which indicates a Varigon plasma-induced change in the chemical composition of the surface.

Under a microscope, clear changes were observed in the salts CuCl_2_ and FeSO_4_. After a treatment time of one hour, a transition from initially turquoise green to a reddish-brownish yellow and then further to a dark gray could be observed for CuCl_2_ (Figure 10). FeSO_4_ exhibited a similar color progression. Here, the initially greenish salt changed to a gray color within one hour, which resulted in a significantly lighter gray tone (Figure 11) than with CuCl_2_. Furthermore, it was noticed during the preparation of the microscope images that yellowish areas had developed from the light-gray surface shortly after the plasma treatment. These areas grew in size after treatment with time until the whole salt had taken on this light-yellow hue after about 8 h. Due to the liquefaction of the FeCl_3_ crystals after treatment, no significant images of these samples were possible to obtaindirectly after the plasma reduction experiments, as only a detachment of the crystals from the solid compound into the formed liquid phase could be observed.

These discolorations are indicative of the formation of different copper oxide species. Usually, copper(II) oxide (CuO) appears with a black coloration and copper(I) oxide (Cu_2_O) has a reddish-brown coloration. This color depends on the particle or crystal size; the larger the crystals of Cu_2_O, the more reddish they appear; however, if the particle size is smaller, Cu_2_O appears yellowish brown [29]. Additionally, the often-stated black color of CuO [30] is not always observed since an anthracite-gray coloration is quite possible with this compound, and this is easily recognizable by the microscope images. It seems counterintuitive at first why more oxide species form in a reductive Varigon plasma with an increasing treatment time. Possible reasons for this could involve the water content in both the crystalline material and in the process gas used. Due to the additional energy input of the plasma discharge, it would be conceivable that oxygen species bind to the metal ions as a result.

The light gray discoloration of FeSO_4_ can be explained by an initial reduction, possibly even partial, to elemental iron. This would have resulted in a partial reoxidation due to the simultaneous plasma activation during treatment and the aeration process after treatment. Contact with oxygen through the aeration process could possibly have disposed the reduced surface fractions of the crystals to oxidize to Fe^3+^ compounds, resulting in the plasma only having significant effects at the surface due to the low penetration depth of 1–2 nm at the maximum [31] of the plasma discharge. Thus, even without an additional oxygen supply, an oxygen input through crystal water inclusions would also be possible, analogous to the assumption for CuCl_2_. Another explanation for the color change could be redox reactions between the outer plasma-treated crystal layer and the crystal interior (which is largely unaffected by the discharge), caused by the potential difference in the varying oxidation states. Thus, a mixed fraction consisting of, for example, a roemerite-like material, could have formed. This would also explain the subsequent yellow discoloration of the crystal material.

Since a plasma treatment only directly affects the upper atomic layers of the materials, further redox reactions occur only after the plasma treatment. A change in the oxidation state first leads to the formation of a reduced outer layer, while the underlying crystal material remains unchanged, and thus two metal compound systems of different oxidation states come into direct contact. This initial situation will lead to further redox reactions that have a lasting effect on the plasma-treated outer layer. In addition, the plasma treatment also causes surface activation, so that atmospheric components can subsequently react with the plasma-activated surface. Therefore, the next research step was carried out with metal salt thin films applied to glass slides. In this way, the plasma treatment was intended to affect the entire layer system as far as possible in order to avoid the formation of different layers with different oxidation levels.

In conclusion, the effect of low-pressure plasma on crystalline and powdered materials was more pronounced than on metallic surfaces. The applied plasma was able to affect more of the material due to the increased surface of smaller particles.

### 3.3. Evaluation of the Reduction of the Low-Pressure Plasma-Treated Metal Salt Layers by XPS

The XPS measurements of the plasma-treated metal salt samples provided information about the chemical composition of the metal salt layers on the glass slides. As indicated in the previous section, CuCl_2_ and FeCl_3_ layers were deposited on glass coverslips and plasma-treated with Varigon gas as the process gas in the time periods described at the beginning.

Within 45 min after plasma exposure, the specimens were transferred to the vacuum lock of the monochromatized X-ray photoelectron spectrometer Phi VersaProbe II (Ulvac-phi, Inc., Osaka, Japan). The system was equipped with an Al-K_α_ source at a photon energy of 1486.6 eV and was operated at a base pressure of 2 × 10^−6^ Pa. The system measures the Ag 3d_5/2_ peak with a FWHM of 0.6 eV at a pass energy of 23.5 eV. Active charge compensation was applied during all measurements with a cool cathode electron flood source and low-energy argon ions. All measurements were conducted at room temperature. The X-ray power was set to 25 W with a beam size of 100 µm. The electron take-off angle was kept constant at 45°. A constant analyzer energy mode was applied with a pass energy of 23.5 eV for high-resolution spectra. Data processing and analysis were conducted with the software package MultiPak v9.9.0.8 (Ulvak-phi, Inc., Osaka, Japan). The energy scale was corrected in the usual manner by using the advantageous carbon peak at 284.8 eV as a charge reference. Peak fitting analysis was achieved using Voigt profiles set at 70% as a lower limit up to 100% Gauss’, and a Shirley background was subtracted from all spectra.

**Figure 12:**
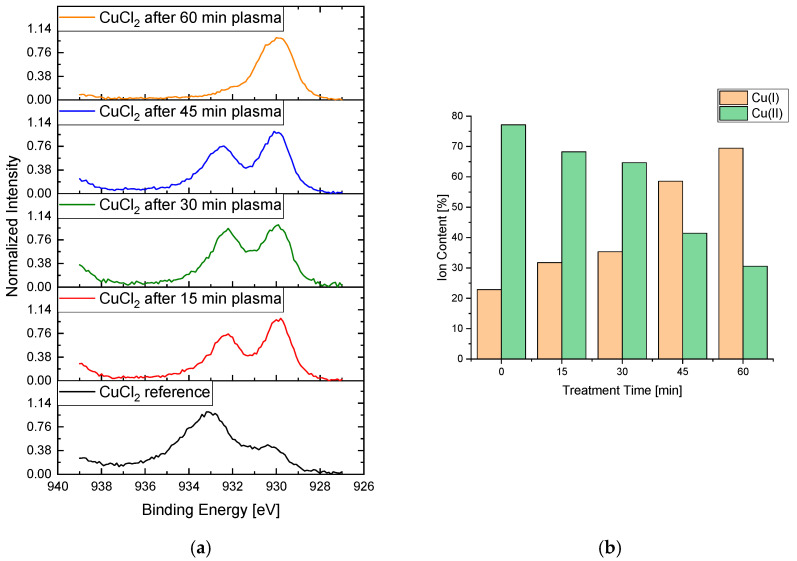
Plot of the respective Cu-2p*_3_* signal (**a**) of copper(II) chloride after 15 (red), 30 (green), 45 (blue), and 60 min (orange) treatment time at a low pressure with Varigon plasma compared to the reference (black). The stacking of these graphs clarifies the decrease in the left Cu^2+^ signal and the increase in the Cu^+^ signal with higher treatment times. The right picture shows a plot of the conversion of Cu^2+^ to Cu^+^ (**b**) at different treatment times, which illustrates a time-dependent reduction trend.

Initially, the analysis of FeSO_4_ did not yield any results, as almost exclusively carbon signals were detected in the analysis, and no iron signals were recorded. The analysis of the copper samples (Figure 12) made visible a time-dependent trend, namely that the reduction fraction increases steadily with an increasing treatment. This is represented in a Cu^+^ signal at approx. 932 eV [32]. A similar decrease in the Cu^2+^ signal at approx. 935 eV [33] could also be observed. Thus, the composition of the copper signal for the reference is approximately 77% Cu^2+^ and 23% Cu^+^. Already after 15 min of Varigon plasma treatment, there was a clear drop in the Cu^2+^ content by 9% to a total of 68%. The Cu^+^ content also increased to the same extent. The most significant change was at the transition from 30 to 45 min of treatment time. A drop from 65% (30 min) to 42% (45 min) in the Cu^2+^ signal was observed. After one hour of treatment time, the ratio between the Cu^2+^ and Cu^+^ signal content had almost reversed compared to the reference, with 31% (Cu^2+^) to 69% (Cu^+^).

**Figure 13:**
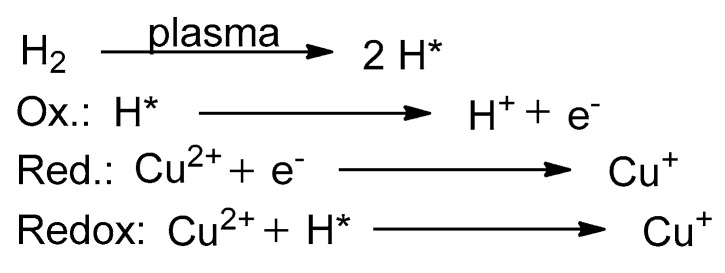
Redox equations of the elemental processes during the plasma reduction of the copper salt layer with the specific oxidation and reduction equations in the second and third line. The first line shows the plasma-induced dissociation of molecular hydrogen to excited atomic hydrogen (H*) and the last stands for the complete redox process.

One essential factor of the plasma’s influence is the generation of atomic hydrogen from its molecular form. Sabat et al. [23] concluded from their research results that the reduction impact and success at low temperatures heavily rely on the amount of atomic hydrogen in the reduction system. The more atomic hydrogen is created, the better the reduction performance. Figure 13 illustrates the whole process with the basic equations. First, plasma dissociates molecular hydrogen. Then, the hydrogen atoms lose their electrons (oxidation), while the copper ions absorb these and change their oxidation state from +2 to +1 (reduction).

**Figure 14:**
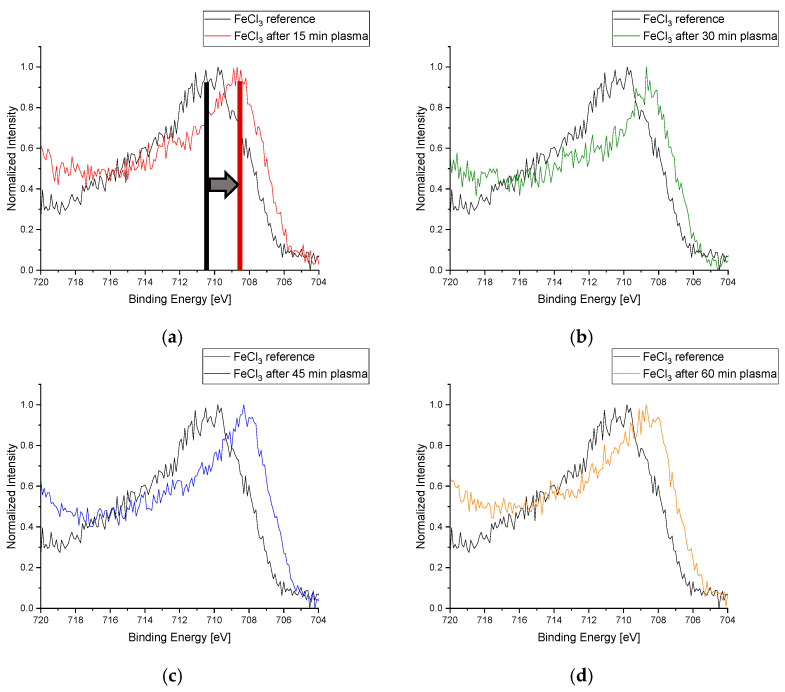
Plot of the respective Fe-2p*_3_* signal of iron(III) chloride after 15 (**a**), 30 (**b**), 45 (**c**), and 60 min (**d**) treatment time at a low pressure with Varigon plasma compared to the reference (black signals in the plots). The clearly visible shift of the plasma-treated samples from the reference sample to lower binding energies proves the plasma-reduction effect on FeCl_3_ layers. The color columns in picture (**a**) visualize the peak positions before (black column) and after plasma treatment (red column) to illustrate the peak shift (arrow).

The initial experiments for the reduction of FeCl_3_ were not successful and no low-pressure plasma effect was observed, except for in one sample. The treatment with Varigon in this case was polluted with ambient air residues due to an incomplete evacuation of the chamber. Based on this observation, a slightly modified procedure was used in the further plasma treatment of the iron samples. After additional samples were prepared, they were dried under a low pressure in the same way as the previous ones and then treated with an oxidative ambient air plasma for one hour before the Varigon plasma was ignited for reduction at the times described previously. XPS analysis of the samples showed a shift in the iron signal to lower binding energies compared to the Fe^3+^ reference for all four treatment durations. This indicates that reduced Fe^2+^ was also formed in the FeCl_3_ samples. Typically, Fe^3+^ compounds have a binding energy of about 710 eV and Fe^2+^ compounds are at about 708 eV [34], which can also be seen in the signals from the 2p_3_-orbitals of the iron of the measured samples (Figure 14).

In contrast to the CuCl_2_ samples, these results showed that a reductive Varigon plasma alone is not sufficient to reduce FeCl_3_. The reductive success through the pre-treatment of an ambient air plasma suggests that an intermediate product was initially formed by this plasma. Partial oxygen-containing ligands could have been formed in the FeCl_3_ compounds. Hereafter that, it would be possible that a chloride ligand was exchanged for a hydroxyl group. This hydroxyl ligand could then have been removed by the hydrogen part of a Varigon plasma as water, while the electrons of the plasma reduced the Fe^3+^ ions to Fe^2+^ ions.

**Figure 15:**
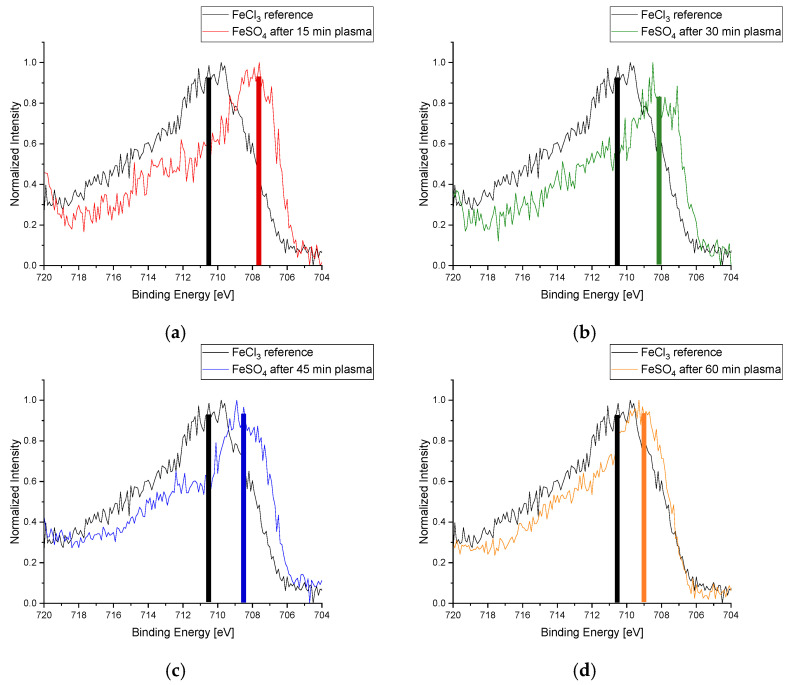
Plot of the respective Fe-2p*_3_* signal of iron(II) sulfate after 15 (**a**), 30 (**b**), 45 (**c**), and 60 min (**d**) treatment time at low pressure with Varigon plasma compared with the reference (black signals in the plots). Similar to the FeCl_3_ samples, a successful reduction can be seen in these spectra by the shift of the plasma-treated samples to lower binding energies. It is noticeable that the shift consistently decreases with a higher treatment duration. The color columns in the pictures visualize the peak shift after plasma treatment compared to the reference (black column).

While the metal chloride layers were formed from a solution in methanol, no iron could be found in the XPS analysis of comparably produced FeSO_4_ layers. For this reason, FeSO_4_ layers were produced from aqueous solution and used for the further experiments and analyses presented. In the case of FeSO_4_, a conversion to Fe^3+^ was already detected in the XPS measurement in the reference sample without a prior oxidative plasma treatment. Oxidation effects seem to have already happened during the application of the metal salt layer due to solvent influences. It is known that in aqueous solutions, FeSO_4_ oxidizes to basic iron(III) sulfate (Fe(OH)SO_4_) under the influence of oxygen. Therefore, the iron was assumed to be in Fe^3+^ state for these samples already after the coating was applied to the cover glasses. These spectra were also compared with the untreated FeCl_3_ sample as representative of Fe^3+^ compounds. Nevertheless, in contrast to the first low-pressure plasma-reduction experiment with FeSO_4_, the adapted process control led to partial reduction successes similar to those of FeCl_3_, as can be seen in Figure 15. It can be concluded that the preparatory plasma oxidation method allows the subsequent reduction success of Fe^3+^ compounds at lower energies and temperatures than would normally be necessary. Another appreciable result can be observed in the spectra of Figure 15. There is a constant decrease in the reduction effect, represented by the less-pronounced shift of the sample peaks from the reference with an increasing plasma-treatment time. A possible explanation for this behavior can be found in the plasma activation processes. The longer the plasma treatment’s duration, the higher the activation impact on the surface. The reduced samples had been transported inside a desiccator under argon atmosphere, but were exposed to ambient air during XPS preparation and the transport to the airlock of the XPS unit. The oxygen of the ambient air was able to partially oxidize the metal salt layers depending on their treatment time due to the higher activation effect of the longer-treated samples. One way to prevent oxidizing compounds from contaminating the reduced layer materials is to work entirely within an inert gas atmosphere—in a glove box for example—until the activation effect vanishes. Another possibility is to keep the reduced materials within the coating chamber under reduced pressure and to coat them directly after the plasma treatment with a sealing parylene barrier layer. It can also be helpful to add lesser metals to the reduced-layer materials as a galvanic anode.

**Figure 16:**
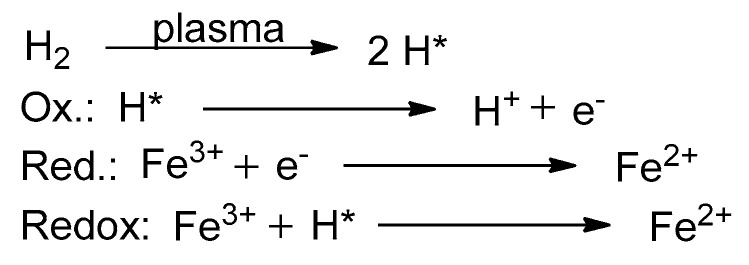
Redox equations of the elemental processes during the plasma reduction of the iron salt layers with the specific oxidation and reduction equations in the second and third line. The first line shows the plasma-induced dissociation of molecular hydrogen to excited atomic hydrogen (H*) and the last stands for the complete redox process.

With reference to Figure 13, it can be assumed that the reduction process of the iron chloride and sulfate layers follows similar principles. The influence of the air plasma during the pre-treatment could not be observed in the available data, but as shown in Figure 16, atomic hydrogen possibly plays a key role as a reducing agent. The iron ions at the surface of the salt layers changed their oxidation state during the reduction from +3 to +2, as can be seen in the experimental data for both FeCl_3_ and Fe(OH)SO_4_.

In summary, the XPS data prove that it was possible to reduce copper(II) with its redox status of +2 and a 3d^9^ electron configuration to its energetically more stable form of copper (I) with an oxidation number of +1 and a 3d^10^ electron configuration. By incorporating an ambient air plasma pre-treatment, it was also possible to convert the iron(III) ions (oxidation state of +3) from their more stable 3d^5^ configuration into the energetically less favorable 3d^6^ configuration of iron(II) with a redox status of +2. As a result, it was not only possible to reduce a noble metal such as copper to a more favorable oxidation state, but also a base metal such as iron, with a lesser electron affinity compared to copper, was able to be reduced to a less advantageous energetic state.

Finally, an effective low-pressure plasma reduction inside a coating device was successful on all the oxidized materials of copper and iron.

## 4. Conclusions

This study investigated the plasma effect of low-pressure microwave plasma generated in a parylene coating device on the modification of the oxidation state of the transition metals iron and copper. The findings can be summarized as follows:

- Microwave ambient air plasma oxidized copper strips, while steel strips remained unchanged.
- CuCl_2_ and FeSO_4_ crystals changed significantly with a Varigon plasma treatment.
- A time-dependent reduction trend with Varigon plasma was observed on CuCl_2_ layers with a clear reduction from Cu^2+^ to Cu^+^ → hydrogen-containing microwave plasma reduced copper-salt layers.
- The reduction of base metal salt layers (FeCl_3_ and FeSO_4_) in a Varigon plasma was successful by implementing an ambient air plasma pre-treatment → metal compounds with a lower standard electrode potential compared with hydrogen were reduced by hydrogen-containing plasma.
- Surface activation effects and the influence of ambient air after treatment caused the reduction impact to decrease in the FeSO_4_ layers.
- The plasma treatments succeeded at low temperatures between 30 °C and 50 °C.

This procedure enables a plasma-induced variation in the oxidation state in thin layers. It also makes this process possible to be included within a parylene coating device to create polymer–metal multilayer structures with defined oxidation states of base and noble metals. Such systems can be used for many applications, for example, catalytic metal surfaces on parylene substrates, which can be applied in difficult geometries. These materials can also serve as UV-protective coatings for sensitive materials or as high refractive layers by incorporating suitable metals.

In comparison to other research attempts, the reduction of oxidized metal compounds was focused on metal salt solutions, which were treated with an atmospheric-pressure plasma source to synthesize metal nanoparticles [15,16,17,18,19,21]. There have also been attempts to create nanoparticles from elemental materials at a low pressure [13,20]. The studies of Crowther et al. [25] and Sabat et al. [23] are very close to this work in terms of using microwave plasma at low pressures. Both examined the reductive plasma effect on oxidized metal compounds, such as iron oxides, silver nitrate patina, and palladium acetate patina. While Crowther et al. focused on noble metals such as silver and palladium, Sabat et al. examined the hydrogen-containing plasma influence on the base metal iron. This study combines the two approaches, using copper materials for noble metals and iron materials as representatives of base metals. Another important difference from this research is the plasma device. While the plasma device in this study is a tool included in a commercially available parylene-coating system, mostly custom-built and individually constructed laboratory devices were used in previous studies.

One limitation of this procedure is the applicability of metal salt thin layers in the nanometer scale due to the low penetration depth of the plasma. The application of low-pressure plasma also limits the size and number of samples to the available space in the treatment chamber. This process was designed for a parylene-coating device and therefore is bound to a limited parameter setup that could be realized in a parylene-coating chamber. These parameters include temperatures up to 100 °C and generator power of 1200 W at the maximum. Microwave plasma also has a very low physical impact on the substrates, and the treatments are dominated by chemical processes.

Future research areas involve examining the reaction mechanism of these plasma-enhanced redox processes. Another aspect for research could be to apply the reduction procedure introduced in this study to other base metal salts, such as zinc or cobalt salts. The aptitude of the suggested applications mentioned earlier also merits further investigation.

## Figures and Tables

**Table 1:** A brief overview of the literature.

Reference	Category	Key Message
[1,2]	Basic principles of the redox state of iron and copper	Iron and copper exist in a variety of oxidation states in nature. The most prominent state for iron is iron(III) and for copper copper(II).
[3,5,9,10]	Parylene–metal multilayer system creation for semiconductor structures	Plasma was used to deposit conducting metal structures in nanometer scale within a semiconductor unit (silicon wafer, interposer, and CMOS) via sputter or beam deposition processes.
[4,6,7,11]	Parylene–metal material compounds for medical implants	Implants for electric conduction purposes and the increase in fracture resistance were designed from parylene metal multilayer systems. The deposition of metals was realized using sputtering processes. The multilayers are intended for use in retinal, brain, and dental implants.
[8,12]	Thin metal layers for electronic devices in combination with parylene layers (for references [8,9])	Metal oxides (Ta_2_O_5_, FeO_x_) as examples of oxidized metals are included in electrical devices for electrowetting and ferromagnetic layers to manipulate exchange bias.
[13,14,15,16,17,18,19,20,21,25,26,27]	Plasma as a tool for the redox state alteration of metal compounds and thin films	Nanoparticles of various metal compounds (TaC, TaN, Ag, Au, Ir) were mostly synthesized from metal salt solution or directly from the elemental state by the application of plasma technology. This technology also causes reductive changes in thin films of oxidized metals.
[22,23,24]	Examination of the plasma mode of functioning on several metal compounds	A mixture of argon and hydrogen gases causes the highest change in aluminum surfaces. Atomic hydrogen provides a better plasma reduction effort, and the plasma influences solved metal salts via the generation of hydrogen peroxide, hydroxyl radicals, and hydrated electrons.

**Table 2:** Composition of the metal strips before and after low-pressure plasma treatment with ambient air for 60 min. These data were obtained from XPS measurements.

Sample	Cu 2p_3_ [at.%]	Fe 2p_3_ [at.%]	O 1s [at.%]	C1s [at.%]
Cu (reference)	4.12 ± 0.55		15.79 ± 2.01	80.09 ± 2.10
Cu (air plasma)	61.80 ± 17.80		20.99 ± 10.27	17.21 ± 8.38
Fe (reference)		0.23 ± 0.27	13.62 ± 2.66	83.90 ± 3.34
Fe (air plasma)		10.35 ± 1.21	65.56 ± 3.23	14.68 ± 1.95

## Data Availability

The data presented in this study are available on request from the corresponding author. The data are being used in a Ph.D. thesis in preparation and are therefore not publicly available.
